# Development of an online intervention for the Rehabilitation Exercise and psycholoGical support After covid-19 InfectioN (REGAIN) trial

**DOI:** 10.3310/nihropenres.13371.1

**Published:** 2023-02-23

**Authors:** Stuart Ennis, Peter Heine, Harbinder Sandhu, Bart Sheehan, Joyce Yeung, David McWilliams, Christina Jones, Martin Underwood, Julie Bruce, Kate Seers, Gordon McGregor

**Affiliations:** 1 Clinical Trials Unit, University of Warwick, Coventry, UK; 2 Cardiopulmonary Rehabilitation, University Hospitals Coventry & Warwickshire NHS Trust, Coventry, UK; 3 The Oxford Psychological Medicine Centre, Oxford University Hospitals NHS Foundation Trust, Oxford, UK; 4 Centre for Care Excellence, Coventry University, Coventry, UK; 5 ICUsteps Peer Support Charity, ICUsteps Peer Support Charity, London, UK; 6 Health Sciences, University of Warwick, Coventry, UK; 7 Centre for Sport, Exercise and Life Sciences, Coventry University, Coventry, UK

**Keywords:** rehabilitation, long-COVID, physical activity, online support, intervention development, physical activity

## Abstract

**Background:**

Up to half of people hospitalised with COVID-19 report diverse and persistent symptoms affecting quality of life for months and sometimes years after discharge (long-COVID). We describe the development of an online group exercise and behavioural support intervention for people who continue to experience such physical and/or emotional health problems more than three months after hospital discharge.

**Methods:**

Intervention development was informed by the Medical Research Council framework for complex interventions. Our multidisciplinary team of academics, clinicians, and people with long-COVID, had collective expertise in the development and testing of complex interventions. We integrated a bio-psycho-social model of care drawing on rehabilitation literature for long-term health conditions and experiences from our pre-pilot study. Multiple stakeholder meetings were held to refine the intervention which was designed to be deliverable within the UK National Health Service. We adhere to TIDieR guidance for transparent and explicit reporting of telehealth interventions.

**Results:**

The final REGAIN online exercise and behavioural support intervention consisted of an initial 1:1 consultation with a trained practitioner, followed by eight online group exercise, and six group support, sessions delivered over eight weeks. Participants could also access an online library of on-demand exercise and support videos.

**Conclusions:**

The final REGAIN intervention, combining exercise and behavioural support, is fully manualised with clear pathways to delivery and implementation. It is currently being tested in a randomised controlled trial. The intervention, developed with extensive patient and stakeholder engagement, could be incorporated into existing NHS rehabilitation programmes, should it prove to be clinically and cost-effective for people with long-COVID.

**Trial registration:**

International Standard Randomised Controlled Trial Number (ISRCTN) 11466448: Rehabilitation exercise and psychological support after COVID-19 infection: REGAIN.

## Plain English summary

Long-COVID has many debilitating symptoms, such as breathlessness, muscle weakness and fatigue, which significantly affect peoples’ physical and mental health and quality of life. Rehabilitation programmes can help people improve their quality of life in other medical conditions with similar symptoms. We developed a programme of physical and mental health rehabilitation specifically to support people with ongoing long-COVID symptoms more than three months after hospital discharge. The programme was developed by people with long-COVID along with clinicians and researchers. The programme described in this article is now being tested in a large research trial to see if it can help people with long-COVID.

## Introduction

‘Long-COVID’ is the term commonly used to describe symptoms which continue or develop after the initial acute infection with COVID-19 has resolved. It includes ongoing symptomatic COVID-19 (from 4 to 12 weeks) and post-COVID-19 syndrome (12 weeks or more), where symptoms cannot be explained by an alternative diagnosis ^1^. As of January 2023, there were approximately 2.1 million people (3.3% of the population) self-reporting long-COVID in the UK ^1^. Of those, 57% reported symptoms lasting at least one year, and 76% reported symptoms that adversely affected their daily activities ^1^. The most common symptoms were fatigue (71%), difficulty concentrating (‘brain fog’) (49%), breathlessness (47%), and muscle aches (46%) ^1^. Symptoms vary in severity within and between individuals, and commonly remit and relapse over time leading to considerable distress and a negative impact on mental wellbeing ^2^. People often feel dismissed by medical professionals, and abandoned after hospital discharge, and commonly seek validation and support elsewhere via peer behavioural support groups and social media ^3, 4^. Long-COVID can have devastating psychological, physical and cognitive consequences that disrupt lives and livelihoods ^5^.

To address long-COVID, an NIHR review ^6^ suggested the need for a holistic, integrated treatment approach rather than symptom by symptom management. This was supported by UK COVID-19 guidelines which recommended pragmatic treatment for psychological, emotional, and physical health ^7^. However, for people with long-COVID, direct face-to-face contact with medical professionals in general, and specifically specialist post-COVID-19 clinics, has been difficult to access, and provision is highly variable. One method of countering these problems is to provide resources online.

The primary digital resource for people living with long-COVID in the UK is ‘Your COVID Recovery’ ^8^ produced by the National Health Service (NHS). ‘Your COVID Recovery’ is a comprehensive NHS online resource providing self-management symptom advice and support. However, this resource lacks the social connection that patients often desire, is necessarily generic, and may not be versatile enough to meet the needs of everyone with long-COVID. Furthermore, awareness of the website is not widespread, and evaluation of patient experience of the intervention has not yet been published.

Currently, few studies have examined rehabilitation for people with long-COVID, particularly in an online setting, although there are reports that multidisciplinary rehabilitation can improve symptoms in long-COVID ^9^ and enhance quality of life for people living with other long-term health conditions with similar symptom profiles, such as Chronic Obstructive Pulmonary Disease (COPD) ^10^ and Severe Acute Respiratory Syndrome (SARS) ^11^. Therefore, a large-scale trial to test such an intervention suitable for online, remote delivery, is required.

This manuscript describes the design and development of an online exercise and behavioural support intervention for people with long-COVID. The REGAIN randomised controlled trial (RCT) was funded by the National Institute of Health Research COVID-19 Recovery and Learning Programme (NIHR132046) to evaluate the clinical and cost-effectiveness of an online exercise and psychological support intervention for people hospitalised with COVID-19 who still reported symptoms more than three months after hospital discharge ^12^.

## Methods

### Ethical approval

Ethical approval was received from the East of England-Cambridge South Research Ethics Committee (REC: 20/EE/0235).

Written informed consent for publication of the participants details and/or their images was obtained from the participants.

### Overview of the development process

**Figure 1.**
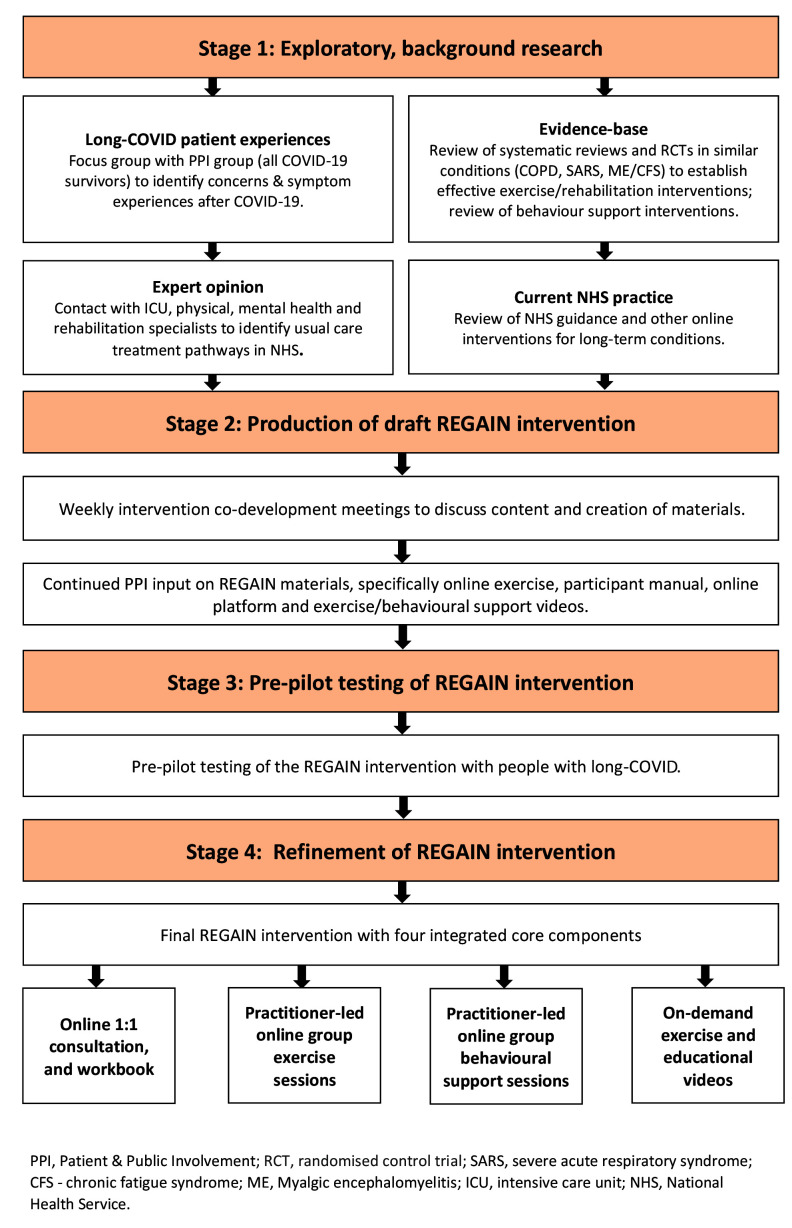
Intervention development process.

We followed the Medical Research Council framework ^13^ for design of complex interventions ( Figure 1). Our manuscript adheres to the recent Template for Intervention Development and Replication (TIDieR) for ‘telehealth’ complex interventions ^14^. 

### Stage 1: Exploratory, background research

A search of bibliographic databases (MEDLINE, PsycInfo and CINAHL) identified literature relating to behavioural or self-management needs for people living with long-COVID. Due to the relative paucity of long-COVID research, we also searched for literature on chronic fatigue syndrome (CFS), chronic obstructive pulmonary disease (COPD), and the 2002–2004 SARS epidemic. Collectively these conditions exhibit several of the most common and debilitating symptoms of long-COVID (e.g. fatigue, lethargy, breathlessness, muscle weakness, brain fog).

Titles and abstracts of papers were assessed for relevance, prioritising recent systematic reviews of needs and interventions in long-COVID, CFS, COPD and SARS. Articles were examined to ensure that the needs identified by our stakeholder groups were addressed by research evidence where possible. Reports of interventions for conditions with similar symptoms helped to identify core components that may be effective for people living with long-COVID.

The literature review revealed limited evidence regarding the efficacy of rehabilitation interventions for COVID-19 survivors ^9^. One RCT (n=72) of respiratory rehabilitation reported improved respiratory function, quality of life (QoL) and anxiety in elderly people with COVID-19 ^15^, but there was little other data available. In other similar conditions, a meta-analysis of 65 RCTs involving 3822 participants reported that pulmonary rehabilitation programmes, combining exercise with various psychological support and education components, were beneficial in improving health-related quality of life (HR-QoL) and exercise capacity in people with COPD ^10^. Similarly, a meta-analysis of eight RCTs (n=1518) concluded that people with CFS may generally benefit and feel less fatigued following exercise therapy, comparable to the effectiveness of cognitive behavioural therapy (CBT), with no evidence to suggest that exercise therapy may worsen outcomes ^16^. Exercise training was also reported to be effective in improving cardiorespiratory and musculoskeletal fitness in people recovering from SARS, although there was no reported effect on HR-QoL ^11^. Other studies highlighted the persistent mental and physical abnormalities in SARS survivors up to two years post-infection ^17^. Exercise therapy alone, therefore, is likely insufficient to optimise recovery for people with long-COVID.

We concluded that to combat the multiple long-term physical and mental health consequences of COVID-19, a combined physical and psychological rehabilitation intervention was required. In order to make the programme as widely accessible as possible during COVID-19 ‘lock-downs’ and reduced access to healthcare, the intervention was developed to be deliverable online.

### Stage 2: Production of draft REGAIN intervention

To holistically appraise the needs of people with long-term COVID-19 symptoms following hospitalisation, a core intervention development team was formed. The team had specialism and extensive experience in clinical academia, physiotherapy, psychiatry, health psychology, clinical exercise physiology, critical care medicine, complex intervention development and implementation, and online rehabilitation and behavioural support. Six people with symptoms of long-COVID (patient and public involvement (PPI) group) were also involved throughout every stage of the development of the REGAIN intervention. Online meetings facilitated discussions informed by evidence from Stage 1 development. The team met frequently with the objective of identifying and formulating the key components of a complex intervention.

The research team identified key themes and priorities from the literature to form the outline for a series of online discussions held between August and October 2020 ( Table 1). Consensus responses relating to core components for inclusion in the exercise and behavioural support intervention were collated into the following categories: online group exercise classes (frequency, intensity, time and type principles), concerns about post exertional malaise/chronic fatigue, exercise programme progression, behavioural support materials, and the feasibility of delivering the intervention online in a NHS setting to participants with diverse symptomology and varying degrees of information technology (IT) literacy. Findings from the stakeholder meetings are summarised in Table 1. A framework of a draft intervention was finalised for pre-pilot testing.

**Table 1.** Key findings from intervention co-development meetings.

Component	Outcome/consensus
**Online group** **exercise classes**	All agreed that one supervised exercise session per week of 20-60 minutes duration, dependent on fitness level, should be tested for acceptability during the pre-pilot phase. Additional pre-recorded exercise classes planned to provide “on- demand” video material for participants to follow unsupervised at home, up to 2-3 per week. Videos would include a variety of exercises to appeal to all, including: Yoga, Pilates, chair-based and breathing exercise videos. Concerns were expressed by patients that some exercises may not be tolerated by more debilitated patients. The need for robust safety/emergency procedures was also highlighted. People with long-COVID symptoms expressed a loss of confidence about being physically active and anxiety about relapse, also concern about over-exertion. Heavy emphasis on fun over intensity was agreed for first few weeks to promote confidence and adherence. All agreed that light-moderate aerobic exercise (40-70% Heart Rate Reserve, RPE- 11-14) realistic for most participants after familiarisation. Gentle mobility, coordination, and balance exercises would be needed after long sedentary periods for some participants. Clinical exercise physiologists should test acceptability during pre-pilot phase. All Long Covid patients agreed that IT literacy might be an obstacle to accessing sessions for older participants.
**Concern about** **post-exertional** **malaise (PEM)**	Members of trial team met with ME groups to discuss dangers of PEM in the long-COVID population. The word exercise seemed to have negative connotations, assumed to only mean high intensity/ exertion levels which they felt would be detrimental to recovery. Use of term physical activity would be better received. Groups were reassured that (a) intensity would be low-moderate and at the comfort level of the individual and (b) that we would minimise risk of PEM be closely monitoring participants before during and after each session, as well as assessing risk of PEM during initial 1:1 consultation (c) pre-determined ‘graded’ approach adopted in previous trials would not be applied to this population. Specific information was provided to participating clinicians during the training sessions highlighting the need to be aware of possibility of PEM and how to address it and a section on PEM was incorporated into the Practitioner Manual provided to all clinicians taking part in REGAIN.
**Exercise** **progression**	It was agreed that exercise physiologists should produce 8-12 exercise session templates of different levels of intensity for standardisation of delivery across the trial. Classes would follow a circuit format with active recovery between exercises. Point made that quite conceivably a group of very low and high ability participants was likely as practitioners would not always be able to arrange groups based on ability level. Therefore, a great degree of versatility was needed with exercise class design and delivery. People with long-COVID symptoms requested ample rest periods and water breaks. Following warm-up and mobility, duration of exercise should be progressed primarily until at 20 minutes of low-moderate aerobic exercise could be achieved for maximal therapeutic benefit (as tolerated). Researchers requested that exercise physiologists collect ratings of perceived exertion (RPE) for intervention fidelity.
**Behavioural** **support materials**	Mind-body disconnect was mentioned by people with long-COVID symptoms, so they felt it really useful to have an integrated intervention. They felt strongly that it wasn’t simply physical symptoms of fatigue and breathlessness but how this affected their thoughts and feelings. Many topics were deemed useful by patients, especially challenging unhelpful thoughts and anxiety management. Pacing of activities was crucial. Talking to the trained practitioner before and after group exercise sessions in supportive environment was thought to be important to ensure continued adherence and group cohesion. This would also promote peer support. Breathing was difficult for some and so being able to stop, take a breath and relax was important. Reinfection also major source of anxiety/worry. The participant workbook was thought by patients to be a long document, but all content was relevant as this could be read in their own time. The written targets were welcomed (e.g. writing down goals), and making notes during the session was deemed useful. Patients preferred paper copy to an online PDF. They suggested colour-coded sections of the workbook to make it less daunting and tabs to make easy to find section for each weekly session. Suggested workbook format included adding the aims of behavioural support, with short clear statements at the beginning of each section of the workbook.
**Feasibility of** **delivery within NHS** **setting**	Exercise physiologists and health psychologists highlighted potential obstacles to online delivery. These included: the need for a second or ‘co-pilot’ practitioner for safety reasons, IT connectivity , participant retention, risk of busy classes, accurate intensity monitoring and adequate behavioural intervention training for practitioners less experienced with behavioural change techniques. Actions were agreed to mitigate these issues: (1) deliver appropriate training for practitioners led by health psychologist and an experienced clinical research fellow; (2) provide comprehensive reference manuals for participants and practitioners to follow; and (3) ensure time spent with each participant during 1:1 consultation to familiarise with the online platform and IT requirements.

### Post exertional malaise

During the development phase, the trial team were contacted by several organisations representing the Myalgic Encephalomyelitis/Chronic Fatigue Syndrome (ME/CFS) community with concerns relating to the potential harms of unsupervised vigorous exercise for people with long-COVID, in particular the risk of post-exertional malaise ^18^. We recognised the lived experience and expertise within these groups and sought to ensure that REGAIN practitioners were appropriately trained in risks relating to post-exertional malaise. Furthermore, early identification screening measures for post-exertional malaise were incorporated into the intervention protocols, particularly during the initial one-to-one consultation and every subsequent exercise session. Practitioners were encouraged to monitor the progress of participants during and after exercise sessions in order to ensure that exercise intensity and volume were within individuals’ capabilities and did not cause undue persistent fatigue or other problems.

### Stage 3: Pre-pilot testing of the REGAIN intervention

To test acceptability and deliverability, the draft REGAIN intervention was feasibility tested in a pre-pilot study with eight people living with long-COVID. The online exercise and behavioural support sessions were delivered from a community NHS exercise rehabilitation facility (Atrium Health, Centre for Exercise and Health, Coventry). Feedback was gathered on the acceptability and practicality of the intervention from eight participants as well as the healthcare practitioners delivering the intervention. This included comments on the written and online trial materials (e.g. practitioner/participant manuals) and the online platforms used to deliver the intervention. This helped the trial team further refine and develop the intervention before proceeding to the main trial. The key findings from the pre-pilot stage are described in Table 2.

**Table 2.** Main findings from pre-pilot study testing REGAIN intervention delivery.

Component	Finding(s)	Implications for REGAIN Intervention
**IT/online** **issues**	Participants not placing device in adequate position for practitioner to view during exercises. Some older participants had difficulty navigating Zoom/MS Teams, enabling microphone/camera and accessing link to session. Some participants accessed the session through their Smartphone which limited functionality (cannot see rest of group) one person held smartphone throughout, so very jerky & too close up; not possible for practitioner to safely monitor exertion levels or technique.	During 1:1 consultation need to go through set up - test their device and where they will set up the camera etc. Log in to zoom and do test session. Practitioners and ‘co-pilot’ to log in early to group sessions as considerable time needed to let everyone into session. Amend workbook/telephone screening calls - smartphone not good enough unless they can 'cast' to a tv from the phone. Laptops & tablets preferred.
**Progression**	Participants found the progression more acceptable once they had gained confidence in the sessions and felt included in short ’debrief’ post exercise with rest of group.	Class templates amended so that Practitioners can alter the duration of exercises and recovery periods to suit each group. Participants to be asked how they feel the day after the session each week as well as post session to guard against post exertional fatigue.
**Safety**	Participants would frequently talk or make noise throughout, inadvertently interrupting practitioners’ instructions. Occasionally participants would disappear from view- going to toilet/answering door- practitioners unsure if patients unwell.	All participants should be instructed to mute during exercise. Hand signals required to indicate whether easy, ok, too hard and another to say I am unwell/need to stop/etc. Set ground rules and instructions at start of each class requesting participants to signal before they leave screen.
**Very** **debilitated** **participants**	One participant was chair bound in the same session as another with relatively high level of fitness. Practitioners needed assistance from ‘co- pilots’ (via chat function) to adequately monitor all participants.	Need alternative chair-based option for all exercises in session templates. Priority should be given to enjoyment over intensity while participants gain confidence. Frequent water breaks should be given and the emphasis placed on the participant to take responsibility for own exertion level
**One to one** **consultation**	Several participants flagged up for case level mental health disorder on anxiety/depression questionnaire. More questioning during consultation revealed higher level of emotional support needed than planned. Some participants spent longer overcoming IT issues than they did talking about their long- COVID.	Consultations need to be at least an hour in order to introduce website and sign-up process, triage participant for exercise classes and go through IT accessibility/optimal camera position etc. Regular meetings with psychologist planned throughout trial for ongoing advice/support for practitioners during trial.
**Behavioural** **education**	Participants required more support coping with anxiety/avoidance around social interaction and not just physical activity. Goal setting session difficult as most participants experienced frequent setbacks and have unknown timescale of their recovery. Some participants dominated in support session and others barely spoke. Practitioners unsure how long to ‘let it go’ before interrupting. Practitioner also unsure about PTSD issues some participants maybe facing and giving advice outside of their competency.	Additional material included in the participant workbook on negative thought patterns. Practitioners need to approach long COVID differently to traditional rehabilitation for long term conditions with linear improvement each week Health psychologists to do additional training with practitioners regarding managing groups.

### Stage 4: Refinement of the REGAIN intervention

***On-demand exercise and educational videos*.** The intervention team, with the support of our patient partners, developed 14 ‘on-demand’ exercise videos which used a similar format to the live online exercise sessions and were designed to account for all abilities. Lower intensity videos with postural stability and balance exercises were included for people with more pronounced levels of fatigue or disability. Some videos were themed (e.g., shopping, housework) to reintroduce activities of daily living and to add some additional context for those unaccustomed to exercise videos. We also included videos of other exercise modalities (e.g., Yoga, Pilates, and high intensity interval training (HIIT)), which had already been produced by the online physical activity provider (BeamFeelGood) used to deliver the intervention. Finally, five videos providing advice and education on lung function and care, including breathing techniques/exercises (Fit for Surgery team, University Hospital Birmingham) and six ‘mindfulness’ videos (Dr Gail Davies) were produced to give participants additional online resources. All videos were hosted on the online platform via a trial-specific protected user area. Participants were encouraged to access these exercise videos, unsupervised, between weekly group sessions and to progress to performing up to two additional exercise sessions per week as tolerated.

### Practitioner-led online group exercise sessions

A series of 12 templates were designed for the online group exercise sessions. They ranged in modality from low intensity chair-based activity to higher intensity whole body exercise. These formed the basis of all online group exercise sessions and ensured a level of exercise programme standardisation throughout the trial. All the exercises within each template required little or no equipment and were easily modifiable to account for different ability levels within the group. Depending on the group, different exercises were swapped in or out of the session template to cater for the specific needs. Generally, the templates included 7–8 exercises to be completed 2–3 times (i.e., 2-3 circuits). The warm-up and cool down lasted 5–10 minutes each.

## Results

### Overview of REGAIN intervention

**Figure 2.**
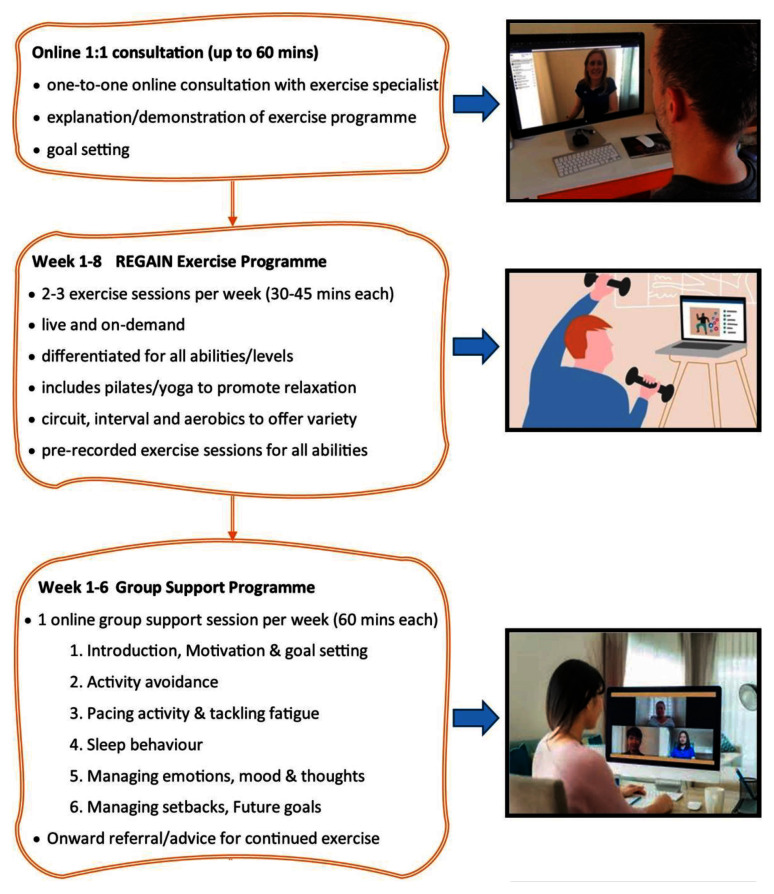
Overview of the REGAIN intervention.

The final REGAIN exercise and behavioural support intervention consisted of four main components ( Figure 2):

1. Online one-to one video consultation and participant workbook
2. Eight-week exercise programme: practitioner-led live online group sessions once per week
3. Six practitioner-led live online group behavioural support sessions (during weeks 1–5 & week 8).
4. On-demand exercise sessions.

### Online one-to-one consultation

The one-to one consultation included several components and was designed to triage the participant for safe exercise, and familiarise them with the IT requirements:

- Clinical history including COVID-19 hospitalisation episode(s), resulting problems and other relevant medical history and co-morbidities
- Potential barriers to participation
- Demonstration of, and sign-up processes, for the online platform.
- Discussion regarding home (e.g. space, lighting) and device set-up for live group exercise and support sessions

### Eight-week exercise programme

The online exercise rehabilitation programme consisted of up to 30 minutes of light to moderate intensity exercise two to three times per week for eight weeks. Practitioners individualised group sessions to accommodate varying abilities, with progressive multi-modality exercise at a manageable intensity, regulated with breathlessness and perceived exertion scales. Participants were encouraged to attend one live online group exercise session each week, and additionally access the online, pre-recorded exercise videos once or twice each week. This equipment-free exercise programme aimed to improve cardiovascular fitness, strength, balance, co-ordination, and confidence. Sessions were undertaken in discrete groups where participants remained in the same group over the eight-week programme.

### Online group support sessions

Emerging evidence highlighted the importance of the biopsychosocial model ^19, 20^ in understanding the interrelationships among risk factors and multidimensional clinical and psychosocial COVID-19 outcomes. The support sessions were based on behaviour change theory (Michie’s COM-B model: mapping key processes and functions to enhance Capability, Opportunity, and Motivation) ^21^, self-efficacy ^22^, motivational interviewing ^23^, and group-based learning ^24^. These theoretical principles were used to inform the psychosocial content, structure and delivery of the sessions in combination with the British Psychological Guidance for management of long-COVID which was available at the time ^25^.

To enhance the psychological capability of participants (as per the COM-B model), support sessions were designed to increase knowledge and understanding about COVID-19 and its impact on daily living. Feedback from patient partners highlighted the need for practitioners to address misinformation from various sources (e.g., friends/relatives, news, social media etc.) which often caused more anxiety and distress. Reduced processing and retention of information due to brain fog and poor concentration also informed the design and content of the support sessions. Resources and activities could be paused at any time and revisited by participants via the provided study materials (PowerPoint presentations, written summaries of key topics, video clips and further reading). Motivation was incorporated into support sessions through exploration of self-identity and the meaning of COVID-19 to the participants and those around them (e.g., family). By discussing COVID-19 experiences in a group setting, participants could see that others were having the same issues and could share experiences and coping strategies. The aim was for participants to feel validated that their symptoms were not only real, but that they were not alone in experiencing them.

Opportunity was the third core principle incorporated into the support sessions: participants were permitted time to engage in sessions in a supportive environment. Group based support sessions were chosen because they can promote behaviour change through shared values and norms, influencing beliefs, and encouraging common motivations and behavioural patterning. We stressed the importance of engaging with all elements of the intervention. To help attenuate fatigue, group support sessions were no longer than 60 minutes. The six group support sessions below are further summarised in Table 3:

**Table 3.** Summary of REGAIN intervention components and theoretical underpinnings.

Intervention component	Aims	Theoretical Framework(s)	Behaviour Change Taxonomy
One to one consultation	To gather relevant information (clinical history) To assess motivations and potential barriers to engagement with the REGAIN Intervention To demonstrate and introduce the online platform To offer an opportunity to answer any questions related to the study or intervention and discuss home environment for engagement with the sessions	COM-B Self-efficacy Communication	Goal setting (behaviour) Restructuring the physical environment Instruction on how to perform a behaviour (online platform) Verbal persuasion about capability (if appropriate)
Support session 1 Introduction, expectations, motivation and goal setting	To introduce and familiarise participant with the programme Getting to know the group Explore personal and group expectations allowing time for planning, prioritising and setting achievable targets Explore motivation, understanding potential short term and long-term benefits of engagement in the programme	COM-B Social Learning Theory Bio-Psycho-Social	Goal setting (behaviour) Goal setting (outcome) Action planning
Group session 2 Fear avoidance and pacing	To introduce relationship between thoughts, feelings/emotions related to avoidance of behaviour (for example fear of aggravating COVID-19 symptoms such as breathlessness/ fatigue) Exploring reasons for fear avoidance and consequences on health outcomes Exploring strategies to overcome fear avoidance and introduce pacing of activities	COM-B Self-Efficacy Bio-Psycho-Social	Information about Antecedents Problem solving
Group session 3 Recovery and sleep, sleep management strategies	To explore how sleep patterns may have changed since having COVID Understanding sleep Introduction of sleep management strategies	COM-B Bio-Psycho-Social	Information about health consequences Problem solving Re-attribution
Group session 4 Management of emotions (perceived stigma, mood/ unhelpful thoughts)	To introduce and explore the impact of COVID on mood, thoughts and behaviour including unhelpful thought patterns Introduce skills for reframing thoughts, encouraging engagement with all components of the programme	COM-B Cognitive Behavioural Principles	Social support (emotional) Information about emotional consequences Monitoring of emotional Consequences Framing/reframing
Group session 5 Understanding stress and anxiety and management strategies	To understand and explore impact of stress on symptom management (acute vs chronic stress) Understand own stress response to COVID and hospitalisation Introduce stress management strategies	COM-B Bio-Psycho-Social	Information about social and environmental consequences Reduce negative emotions
Group Session 6 Managing setbacks and long-term behaviour change and future goals.	To consolidate learning from previous sessions and reinforce long term behaviour change To introduce management of setbacks and strategies of problem solving for long term change	COM-B Group Based Learning Self-management	Problem solving Review behaviour goal(s) Social support (emotional) Comparative imagining of future outcomes Self-talk Focus on past success
Practitioner handbook	To provide step-by-step guidance on delivering the REGAIN intervention To provide facilitation skills and motivational interviewing prompts and questions for group sessions	COM-B Motivational Interviewing	Feedback on behaviour (through quality assessment and observations of sessions) Credible source
Participant workbook	Provide information on programme structure, safety and contact information for further support if needed Provide information and content for each session, with clear aims, and tasks to complete to consolidate learning To allow a log of personal progress and reflection and point of reference during the programme and beyond	COM-B Self-Efficacy Bio-Psycho-Social	Self-monitoring of outcome(s) of behaviour Information about health consequences Behavioural practice/rehearsal
Live group exercise sessions	To increase ability to engage in physical activity To increase strength, flexibility, balance and aerobic capacity. To increase confidence in ability to move and engage in physical activity To encourage physical activity outside of live sessions. To strengthen group dynamics	COM-B Self-Efficacy Group Based Learning Social Learning Theory	Feedback on outcome(s) of behaviour Instruction on how to perform a behaviour Demonstration of the Behaviour Graded tasks Body changes
On demand exercise Videos	To increase ability to engage in physical activity To increase strength, flexibility, balance and aerobic capacity. To increase confidence in ability to move and engage in physical activity To encourage physical activity outside of live sessions.	COM-B Self-Efficacy	Demonstration of the Behaviour Instruction on how to perform a behaviour Behavioural practice/Rehearsal Body changes
Other on demand videos; breathing technique, mindfulness	To provide advice and education on relevant topics. To provide associated skills/behaviours to promote engagement in physical activity To increase confidence in ability to move and engage in physical activity	COM-B Self-Efficacy	Demonstration of the Behaviour Instruction on how to perform a behaviour Behavioural practice/Rehearsal Distraction

Session 1: Introduction, expectations, motivation and goal setting

Session 2: Fear avoidance and pacing

Session 3: Management of emotions (perceived stigma, mood/unhelpful thoughts)

Session 4: Recovery and sleep, sleep management strategies

Session 5: Understanding stress and anxiety, and management strategies

Session 6: Managing setbacks and long-term behaviour change and future goals.

### Behavioural support videos

Feedback from our patient partners and intervention practitioners suggested potential benefit of having a short video or conversation where topics could be explored and viewed during the group sessions. We developed short introductory videos for each of the six group support topics. The videos featured discussions between the trial health psychologist and patient partners, filmed and edited by a professional production company. Practitioners played the relevant video at the start of each group support session with the intention that participants may recognise patterns of behaviour in themselves, thus stimulating support group discussion.

### Intervention fidelity: practitioner training and quality control

To ensure standardisation of intervention delivery, all practitioners completed a full day of training. This included practical and theoretical components, delivered by a clinical exercise practitioner and health psychologist. Guidance was provided on delivery of behavioural education sessions, motivational interviewing techniques, and group facilitation skills. All practitioners completed an assessment after training, to gauge readiness to deliver the intervention and/or the need for further training or support. Practitioners were provided with comprehensive written instruction manuals and met monthly with a senior health psychologist and other members of the intervention delivery team for ongoing support, advice and troubleshooting. All intervention components were within the scope of normal practice for the clinicians involved. The REGAIN intervention was designed for delivery by NHS clinical exercise physiologists or physiotherapists. All practitioners were subject to regular quality control reviews. This ensured that exercise and behavioural support sessions were delivered in a standardised manner. All online sessions were recorded for independent review as part of a separate process evaluation.

### Safety

Supervised sessions were led by practitioners experienced in the assessment, prescription, and delivery of exercise for multi-morbid clinical populations. Pre-exercise session online poll questions were completed by participants to capture any adverse events experienced since the previous supervised session. Practitioners also ended each session by inquiring about any symptoms or adverse events the participants may have experienced. Participants with any issues were asked to remain online after the session or were contacted by telephone or email to explore further. If a participant failed to attend an intervention appointment, the practitioner attempted to contact them via telephone or email to ascertain their welfare.

Exercise carries a very small theoretical risk of complications. All participants were assessed during the one-to-one consultation for any underlying health conditions or severe complications related to COVID-19. Participants were excluded from the study at the eligibility stage where exercise was clearly contraindicated, as assessed by a clinical member of the research team. A further assessment was undertaken by the REGAIN practitioner, through discussion with the patient about their current health, at the time of the initial online intervention assessment. During all group online exercise and support sessions, a second practitioner (‘co-pilot’) was immediately available online to assist with any emergencies, or if a participant became unresponsive or left the session without prior notification.

## Discussion

Regular physical activity can benefit people recovering from illness by increasing cardiorespiratory fitness and muscle strength, and reducing breathlessness. However, overly vigorous or prolonged activity without adaptation for ability or adequate rest periods can be detrimental to recovery and motivation, particularly for people with post-viral fatigue. It is important to mediate the psychosocial obstacles to activity and include intervention components designed to reduce anxiety and improve confidence, thus supporting people to regain physical strength, mobility, and independence. Provision of psychological support has become more challenging due to online delivery and differing levels of IT literacy. Hence, during development and pre-pilot testing, it was consistently demonstrated that our intervention would need to be as accessible and well supported as possible to avoid poor uptake and maintain adherence.

The introduction of an hour-long one-to-one consultation prior to the group sessions, a ‘co-pilot’ facilitator during the exercise sessions, and a comprehensive printed participant workbook aimed to address participants’ concerns about their condition and any additional fear of engaging with digital technology. During piloting, we discovered that not only did the support sessions enhance engagement with the exercise sessions, but that the opposite was also true. We found substantial unmet need for this patient group in being able to talk about their traumatic experiences in hospital with COVID-19, and their need to validate their ongoing problems since discharge. Our patient stakeholders expressed their desire to return to activities of normal living, (e.g., returning to work, walking to collect their children from school), or resume basic domestic chores at home. This required not only improved aerobic endurance and muscle strength, but also the knowledge, confidence and skills to cope with the inevitable peaks and troughs of recovery. Exercise practitioners also expressed their desire to improve their own group facilitation skills to best support the complex emotional needs of patients traumatised by COVID-19 and hospitalisation. The final fully manualised complex intervention for the REGAIN trial was co-produced with patients, lay people and healthcare professionals, and has been rolled out for testing in the REGAIN randomised controlled trial.

## Conclusion

The REGAIN trial will be the largest study to-date to test whether online group exercise rehabilitation and psychological support delivered by NHS professionals is clinically and cost-effective for people with long-COVID symptoms more than three months after hospitalisation, when compared to best practice usual care. The REGAIN intervention, developed with extensive patient and stakeholder engagement, could be incorporated into existing NHS rehabilitation programmes, should it prove to be clinically and cost-effective for people with long-COVID.

## Data Availability

Dryad: REGAIN trial intervention development process evaluation data, https://doi.org/10.5061/dryad.n8pk0p30r ^26^. This project contains the following underlying data: REGAIN_PPI_feedback_on_behavioural_component_and_practitioner_manual.doc Live_online_exercise_session.docx Data are available under the terms of the Creative Commons Zero "No rights reserved" data waiver (CC0 1.0 Public domain dedication).
