# Supplementary material for: Development of an online intervention for the Rehabilitation Exercise and psycholoGical support After covid-19 InfectioN (REGAIN) trial
Source: NIHR Open Res. 2023 Jul 14;3:10. Originally published 2023 Feb 23. [Version 2] doi: 10.3310/nihropenres.13371.2 (PMC10593321; doi:10.3310/nihropenres.13371.2)
Supplement: Peer review history

# Reviewer report: Reviewer response for version 2

**DOI:** 10.3310/nihropenres.14584.r29899

**Published:** 2023-07-28

**Authors:** Stefan Tino Kulnik

**Affiliations:** 1 Ludwig Boltzmann Institute for Digital Health and Prevention, Ludwig Boltzmann Gesellschaft, Salzburg, Austria

I thank the authors for addressing my comments.

 I note that the members of the PPI group have now been named in the acknowledgements, which is a lovely touch.

 (One small correction can be incorporated in the typesetting: there is an incomplete part-sentence in the second paragraph of the discussion which reads "that the opposite was also true" and which can be deleted.)

Is the work clearly and accurately presented and does it cite the current literature?

Yes

If applicable, is the statistical analysis and its interpretation appropriate?

Not applicable

Are all the source data underlying the results available to ensure full reproducibility?

Yes

Is the study design appropriate and is the work technically sound?

Yes

Are the conclusions drawn adequately supported by the results?

Yes

Are sufficient details of methods and analysis provided to allow replication by others?

Yes

Reviewer Expertise:

Digital health, rehabilitation, physiotherapy, co-design, patient and public involvement, complex interventions, behaviour change

I confirm that I have read this submission and believe that I have an appropriate level of expertise to confirm that it is of an acceptable scientific standard.

# Reviewer report: Reviewer response for version 2

**DOI:** 10.3310/nihropenres.14584.r29898

**Published:** 2023-07-28

**Authors:** Garry A. Tew

**Affiliations:** 1 Institute for Health and Care Improvement, York St John University, York, UK

Thank you for updating the manuscript.

Is the work clearly and accurately presented and does it cite the current literature?

Yes

If applicable, is the statistical analysis and its interpretation appropriate?

Not applicable

Are all the source data underlying the results available to ensure full reproducibility?

Yes

Is the study design appropriate and is the work technically sound?

Yes

Are the conclusions drawn adequately supported by the results?

Yes

Are sufficient details of methods and analysis provided to allow replication by others?

Yes

Reviewer Expertise:

Professor of Clinical Exercise Science

I confirm that I have read this submission and believe that I have an appropriate level of expertise to confirm that it is of an acceptable scientific standard.

# Reviewer report: Reviewer response for version 1

**DOI:** 10.3310/nihropenres.14502.r29251

**Published:** 2023-06-02

**Authors:** Stefan Tino Kulnik

**Affiliations:** 1 Ludwig Boltzmann Institute for Digital Health and Prevention, Ludwig Boltzmann Gesellschaft, Salzburg, Austria

Thank you for inviting my peer review of this manuscript entitled „Development of an online intervention for the Rehabilitation Exercise and psycholoGical support After covid-19 InfectioN (REGAIN) trial“. The manuscript describes the stepwise design of a tele-rehabilitation intervention for individuals with persisting symptoms of COVID-19 more than 3 months after infection. Intervention design was guided by the Medical Research Council framework for the development of complex interventions. The reporting followed the TIDieR checklist, including the recent extension for telehealth interventions. 

 I appreciate the detailed description of the intervention development process in this well-presented manuscript, which demonstrates good practice for transparency and scientific rigour and can be helpful for other intervention developers. I have the following comments for the authors‘ consideration, which I suggest to further strengthen the manuscript: 

**In the plain English summary**, you could explicitly state that this is an online/remote intervention. 

**In the Methods:**

 Figure 1 is very helpful. I noticed that there are 4 components described in Stage 1 in Figure 1 (patient experience, evidence base, expert opinion, and current NHS practice), but in the main text under Stage 1 there is description of the review of evidence only. For consistency, I suggest to also briefly describe the other 3 components in the main text under Stage 1.

 It would also be helpful for readers to be provided more information on the make-up of the PPI group (number of participants, gender distribution, age groups, months of living with long COVID), and the number and clinical roles of the individuals who were consulted for their expert opinions. 

 Stage 3 of the development process is described as ‚pre-pilot‘, which could be read to imply that there follows a formal ‚full‘ pilot study – but I understand that the intervention went directly to a definitive trial. Perhaps you can clarify this in the manuscript. 

 Please add a description of the timeline (month/year to month/year), ideally for each of the Stages. 

**In the Results:**

 I enjoyed reading about the many good design decision that were made during the development process, such as the short introductory videos to stimulate support group discussion, and the thorough approach for screening for adverse events. 

 Tables 1, 2, and 3 and Figure 2 provide a good amount of relevant and helpful information. Some sentences seem abbreviated with missing articles, which I feel could be written out for better readability. Acronyms used in the tables could be explained in a legend. In Figure 2, I noticed that the wording of the online group support sessions 1-6 is not completely consistent with Table 3 and the main text. The image to illustrate the exercise programme in Figure 2 could better be an image without exercise equipment, as I understand it was a deliberate design decision to select exercises without need for equipment. In table 3 (row ‚Group session 2‘, last column) the first behaviour change taxonomy seems to be incomplete (‚Information about‘). 

 It was not quite clear to me whether all intervention components are intended to be delivered by clinical exercise physiologists or physiotherapists (i.e., the group support sessions also). You could state this more clearly. 

 Regular quality control reviews and recording of online sessions for independent review are described. Here it would be helpful to have more detail on who conducts these reviews, whether any particular assessment tools are used, and whether this is intended solely for trial purposes or also as an ongoing quality assurance measure if the intervention would be rolled out in practice. 

 I appreciated the design decisions for patient safety. In table 1, there is mention of aiming at a rate of perceived exertion (RPE) of 11-14. You could elaborate on this in the main text also and add a reference to the Borg RPE scale. 

 Table 3 provides a helpful linkage of intervention components with behaviour change theory. I would suggest that adding a figure with an explicit logic model is also required, to give readers an overview of the proposed mechanisms of action, linking through from the context and problem to the anticipated behavioural and clinical outcomes. This would be in line with the MRC framework recommendations. 

 Although the TIDieR checklist is not provided as a separate document, I think the manuscript covers most items of the checklist. Specifically with regard to the tele-rehabilitation/telehealth aspect, you could add details of the technical and data regulatory requirements of the intervention (e.g., is it necessary to use certain video-conferencing tools to meet data protection regulations, etc.). 

**In the Discussion**, 2nd paragraph, the meaning of the statement‚ but that the opposite was also true was unclear to me. 

 I note the two supplemental data files which are available for download on Dryad. These are helpful, but you could augment the files by adding more contextual detail for the reader, such as dates, number and characteristics of persons involved, who moderated/facilitated the activities, etc.

Is the work clearly and accurately presented and does it cite the current literature?

Yes

If applicable, is the statistical analysis and its interpretation appropriate?

Not applicable

Are all the source data underlying the results available to ensure full reproducibility?

Yes

Is the study design appropriate and is the work technically sound?

Yes

Are the conclusions drawn adequately supported by the results?

Yes

Are sufficient details of methods and analysis provided to allow replication by others?

Yes

Reviewer Expertise:

Digital health, rehabilitation, physiotherapy, co-design, patient and public involvement, complex interventions, behaviour change

I confirm that I have read this submission and believe that I have an appropriate level of expertise to confirm that it is of an acceptable scientific standard, however I have significant reservations, as outlined above.

# Response

**Published:** 2023-07-23

**Authors:** Gordon McGregor

**Reviewer 2**

*Thank you for inviting my peer review of this manuscript entitled „Development of an online intervention for the Rehabilitation Exercise and psycholoGical support After covid-19 InfectioN (REGAIN) trial“. The manuscript describes the stepwise design of a tele-rehabilitation intervention for individuals with persisting symptoms of COVID-19 more than 3 months after infection. Intervention design was guided by the Medical Research Council framework for the development of complex interventions. The reporting followed the TIDieR checklist, including the recent extension for telehealth interventions.*

*I appreciate the detailed description of the intervention development process in this well-presented manuscript, which demonstrates good practice for transparency and scientific rigour and can be helpful for other intervention developers. I have the following comments for the authors‘ consideration, which I suggest to further strengthen the manuscript:*

**Thank you for your thorough and insightful review. We have amended the manuscript, figures, and tables accordingly for detail, clarity, consistency and readability (unless otherwise stated) in response to each of your suggestions below.**

*In the plain English summary, you could explicitly state that this is an online/remote intervention.*

*In the Methods:*

*Figure 1 is very helpful. I noticed that there are 4 components described in Stage 1 in Figure 1 (patient experience, evidence base, expert opinion, and current NHS practice), but in the main text under Stage 1 there is description of the review of evidence only. For consistency, I suggest to also briefly describe the other 3 components in the main text under Stage 1.*

*It would also be helpful for readers to be provided more information on the make-up of the PPI group (number of participants, gender distribution, age groups, months of living with long COVID), and the number and clinical roles of the individuals who were consulted for their expert opinions.*

*Stage 3 of the development process is described as ‘pre-pilot‘, which could be read to imply that there follows a formal ‚full‘ pilot study – but I understand that the intervention went directly to a definitive trial. Perhaps you can clarify this in the manuscript.*

**The pre-pilot phase preceded a 3-month internal pilot which continued seamlessly into the full trial.**

*Please add a description of the timeline (month/year to month/year), ideally for each of the Stages.*

*In the Results:*

*I enjoyed reading about the many good design decision that were made during the development process, such as the short introductory videos to stimulate support group discussion, and the thorough approach for screening for adverse events.*

*Tables 1, 2, and 3 and Figure 2 provide a good amount of relevant and helpful information. Some sentences seem abbreviated with missing articles, which I feel could be written out for better readability.*

*Acronyms used in the tables could be explained in a legend.*

*In Figure 2, I noticed that the wording of the online group support sessions 1-6 is not completely consistent with Table 3 and the main text.*

*The image to illustrate the exercise programme in Figure 2 could better be an image without exercise equipment, as I understand it was a deliberate design decision to select exercises without need for equipment.*

*In table 3 (row ‚Group session 2‘, last column) the first behaviour change taxonomy seems to be incomplete (‚Information about‘).*

*It was not quite clear to me whether all intervention components are intended to be delivered by clinical exercise physiologists or physiotherapists (i.e., the group support sessions also). You could state this more clearly.*

*Regular quality control reviews and recording of online sessions for independent review are described. Here it would be helpful to have more detail on who conducts these reviews, whether any particular assessment tools are used, and whether this is intended solely for trial purposes or also as an ongoing quality assurance measure if the intervention would be rolled out in practice.*

**Determination of delivery practices should the intervention be rolled out in clinical practice are out with the scope of this manuscript.**

*I appreciated the design decisions for patient safety. In table 1, there is mention of aiming at a rate of perceived exertion (RPE) of 11-14. You could elaborate on this in the main text also and add a reference to the Borg RPE scale.*

**Reference added.**

*Table 3 provides a helpful linkage of intervention components with behaviour change theory. I would suggest that adding a figure with an explicit logic model is also required, to give readers an overview of the proposed mechanisms of action, linking through from the context and problem to the anticipated behavioural and clinical outcomes. This would be in line with the MRC framework recommendations.*

**Logic model added (figure 3).**

*Although the TIDieR checklist is not provided as a separate document, I think the manuscript covers most items of the checklist. Specifically with regard to the tele-rehabilitation/ telehealth aspect, you could add details of the technical and data regulatory requirements of the intervention (e.g., is it necessary to use certain video-conferencing tools to meet data protection regulations, etc.).*

**Regulatory compliance section added.**

*In the Discussion, 2nd paragraph, the meaning of the statement‚ but that the opposite was also true was unclear to me.*

*I note the two supplemental data files which are available for download on Dryad. These are helpful, but you could augment the files by adding more contextual detail for the reader, such as dates, number and characteristics of persons involved, who moderated/facilitated the activities, etc.*

**These are raw data, thus cannot be altered. Information regarding data collection is outlined in the manuscript.**

# Reviewer report: Reviewer response for version 1

**DOI:** 10.3310/nihropenres.14502.r29247

**Published:** 2023-05-18

**Authors:** Garry A. Tew

**Affiliations:** 1 Institute for Health and Care Improvement, York St John University, York, UK

The manuscript by Ennis and colleagues describes the development of the REGAIN intervention; an online group exercise and behavioural support intervention for people with long-COVID.

 A multi-stage development process was undertaken, including: (1) background research, (2) co-production meetings to draft the intervention, (3) pre-piloting of the intervention with people suffering from long-Covid, and (4) refinement of the intervention based on initial experiences and feedback.

 The final intervention included four main components: (1) an online 1:1 consultation, (2) online group exercise sessions, (3) online group behavioural support sessions, and (4) on-demand exercise and educational videos.

 The manuscript is clearly written and includes everything that I would expect to see for this type of paper. I will be interested to see the results of the trial. I only have two relatively minor suggestions:

1. In figure 2, consider adding something about the on-demand exercise sessions and consider repositioning the information on the behavioural support sessions so that it doesn’t look like it happens after the exercise programme.
2. I was left wanting a bit more information about the content of the group exercise sessions and on-demand exercise videos. Consider providing access to at least one of the 12 exercise session templates via the manuscript. And a link to at least one of the 14 videos.

Is the work clearly and accurately presented and does it cite the current literature?

Yes

If applicable, is the statistical analysis and its interpretation appropriate?

Not applicable

Are all the source data underlying the results available to ensure full reproducibility?

Yes

Is the study design appropriate and is the work technically sound?

Yes

Are the conclusions drawn adequately supported by the results?

Yes

Are sufficient details of methods and analysis provided to allow replication by others?

Yes

Reviewer Expertise:

Professor of Clinical Exercise Science

I confirm that I have read this submission and believe that I have an appropriate level of expertise to confirm that it is of an acceptable scientific standard.

# Response

**Published:** 2023-07-23

**Authors:** Gordon McGregor

**Thank you for these helpful suggestions. We have amended figure 2 accordingly and have added an example exercise session template.**
